# Supplementary material for: Rapid online assessment of reading ability
Source: Sci Rep. 2021 Mar 18;11:6396. doi: 10.1038/s41598-021-85907-x (PMC7973435; doi:10.1038/s41598-021-85907-x)
Supplement: Supplementary file 1 — Supplementary Information. [file 41598_2021_85907_MOESM1_ESM.docx]

**SUPPLEMENTARY MATERIAL**

Rapid Online Assessment of Reading Ability

Jason D. Yeatman*^1,2^, Kenny An Tang*^1,2^, Patrick M. Donnelly^3,4^, Maya Yablonski^1,2,5^, Mahalakshmi Ramamurthy^1,2^, Iliana I. Karipidis^6^, Sendy Caffarra^1,2,7^, Megumi E. Takada^1,2^, Klint Kanopka^2^, Michal Ben-Shachar^5,8^, Benjamin W. Domingue^2^

1. Stanford University School of Medicine, Division of Developmental-Behavioral Pediatrics, Stanford, CA, USA
2. Stanford University Graduate School of Education, Stanford, CA, USA
3. University of Washington, Institute for Learning & Brain Science, Seattle, WA, USA
4. University of Washington, Department of Speech & Hearing Sciences, Seattle, WA, USA
5. The Gonda Multidisciplinary Brain Research Center, Bar Ilan University, Ramat-Gan, Israel
6. Center for Interdisciplinary Brain Sciences Research, Department of Psychiatry and Behavioral Sciences, School of Medicine, Stanford University, Stanford, CA, USA
7. Basque Center on Cognition, Brain and Language, San Sebastian, Spain
8. Department of English Literature and Linguistics, Bar-Ilan University, Ramat-Gan, Israel


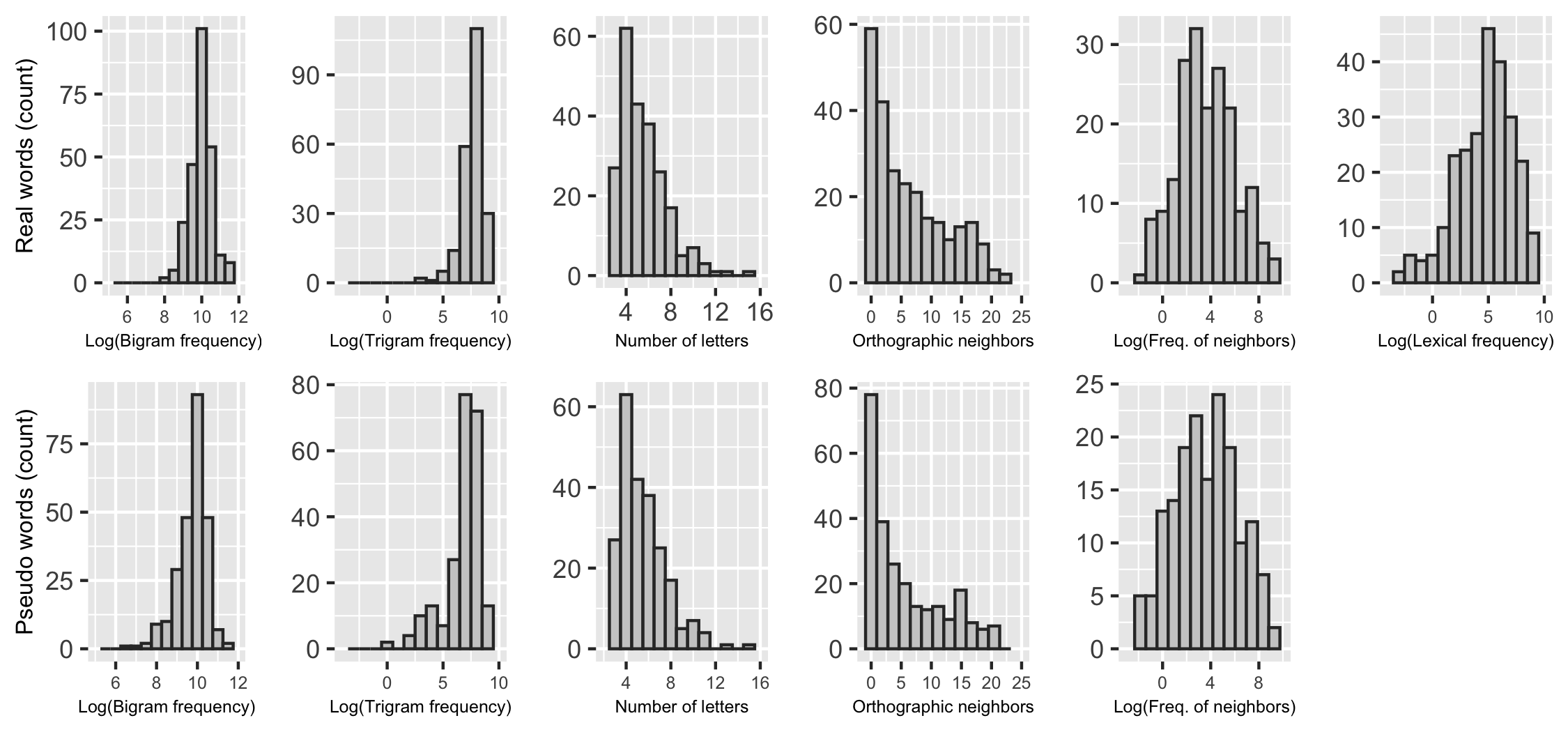


***Supplementary Figure 1. Lexical and orthographic properties of the stimuli.*** *(Top panel) Bigram frequency, trigram frequency, number of letters, number of orthographic neighbors, frequency of orthographic neighbors and lexical frequency for the 250 real words used in the lexical decision task (LDT). (Bottom panel) Bigram frequency, trigram frequency, number of letters, number of orthographic neighbors, frequency of orthographic neighbors for the 250 pseudo words used in the LDT.*

**
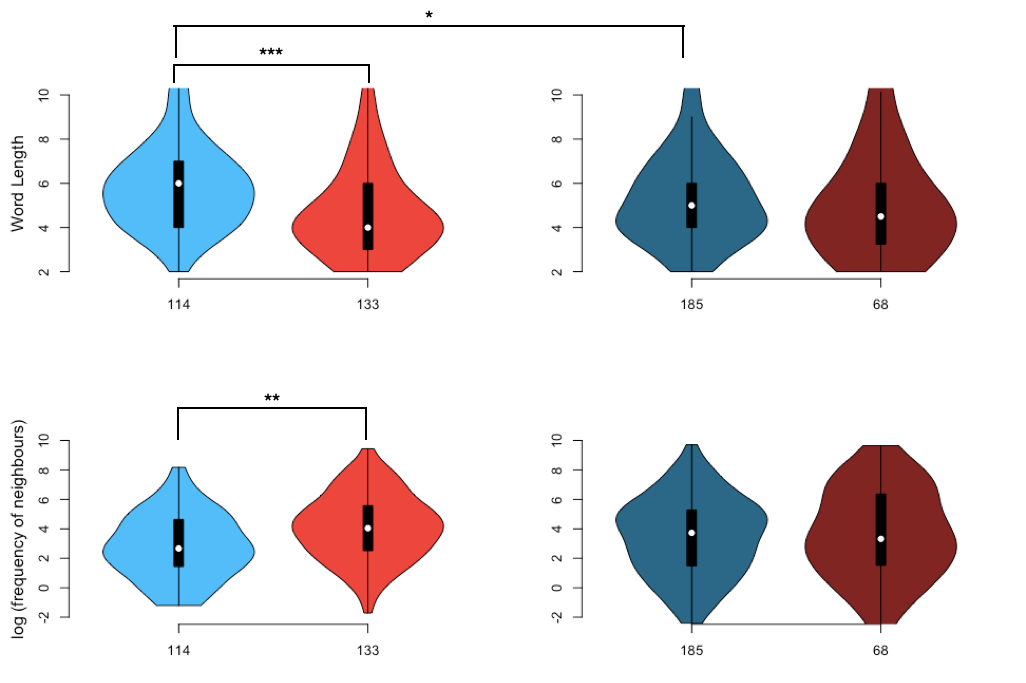
**

***Supplementary Figure 2:*** *Top Panel (left: real words; right: pseudowords): shows the distribution of word length for the retained real words (BLUE) and rejected real words (RED) from step 3 of the IRT analysis. We found that the retained real words were longer than rejected real words (p <0.0001***********) and retained pseudowords (p =0.050*********). Bottom Panel (left: real words; right: pseudowords): Pairwise comparisons revealed that retained real words were significantly lower in log frequency of its orthographic neighbors compared to the rejected real words (p <0.0004**********). These analyses show that the more unique a word is (longer and with low-frequency neighbors) the higher its discriminative power of reading performances. Darker colors are pseudowords and lighter colors are real words.*

**Optimized Stimulus Lists (ROAR *Version 2*)**

|  | **List 1** | **List 2** | **List 3** |
| --- | --- | --- | --- |
| **1** | play | time | him |
| **2** | we | plays | people |
| **3** | miss | moon | open |
| **4** | black | left | look |
| **5** | become | plane | candy |
| **6** | sun | come | morning |
| **7** | shine | toy | contain |
| **8** | liberty | bangs | gravel |
| **9** | however | long | imagine |
| **10** | back | natural | watch |
| **11** | without | farmer | sick |
| **12** | pickle | animal | lame |
| **13** | resolve | jewel | investigate |
| **14** | elements | budget | distance |
| **15** | brought | into | pretty |
| **16** | arm | building | money |
| **17** | will | understand | hot |
| **18** | best | number | chance |
| **19** | custom | went | grasshopper |
| **20** | answer | give | garden |
| **21** | testify | circus | qualify |
| **22** | them | peace | round |
| **23** | waves | truck | agreed |
| **24** | library | wood | more |
| **25** | from | tried | complete |
| **26** | guess | hammer | history |
| **27** | describe | famous | compliment |
| **28** | inside | great | through |
| **29** | purchase | pencil | short |
| **30** | faster | light | almost |
| **31** | mystery | business | question |
| **32** | dryer | pledge | configuration |
| **33** | invent | doubtful | an |
| **34** | reverse | brush | begin |
| **35** | once | street | spy |
| **36** | whose | guarantee | overwhelm |
| **37** | fatter | accustomed | garment |
| **38** | telescoped | potent | appropriates |
| **39** | uoy | hu | ap |
| **40** | fror | ypla | khtna |
| **41** | om | ot | fafever |
| **42** | ujst | asca | cok |
| **43** | inni | spu | orn |
| **44** | wared | sain | swane |
| **45** | leld | shring | runey |
| **46** | arsher | bove | slace |
| **47** | blans | germantia | drustion |
| **48** | ggnoi | unger | alk |
| **49** | bledge | cotplicant | cas |
| **50** | cuppom | clomify | oban |
| **51** | ancrusinishness | charks | pame |
| **52** | crit | ase | hane |
| **53** | ef | jixel | che |
| **54** | ip | ge | twew |
| **55** | cibsus | insows | guoys |
| **56** | slellen | imeyits | phlere |
| **57** | tfaer | mun | daople |
| **58** | trood | roder | pealt |
| **59** | losh | benon | rebosks |
| **60** | loms | yos | utashing |
| **61** | nied | lant | cotosks |
| **62** | sint | baafness | albust |
| **63** | beeltisul | destrifs | duttery |
| **64** | telechinct | wase | banny |
| **65** | sudsage | speveal | collosks |
| **66** | bulbet | mich | molent |
| **67** | fattify | fite | liffs |
| **68** | hus | besime | brubs |
| **69** | groon | mannanency | freat |
| **70** | strosh | mout | woft |
| **71** | coth | sids | fistory |
| **72** | nop | flankfopper | scays |
| **73** | trull | ablood | naw |
| **74** | hust | complase | snose |
| **75** | streat | shink | chanding |
| **76** | bads | mader | rackle |
|  |  |  |  |
| **77** | sit | fun | hello |
| **78** | listen | cold | name |
| **79** | lunch | bathroom | good |
| **80** | night | teacher | hungry |
| **81** | tis | nuf | loleh |
| **82** | stenil | dolc | eamn |
| **83** | nulch | throomba | dogo |
| **84** | ginth | chareet | gurynh |

***Supplementary Table 1. Stimuli for ROAR version 2 used in Study 2.*** *76 items (38 real words, 38 pseudowords) were selected based on the data from Study 1. Eight new items (4 real words, 4 pseudowords) were added to each list to include more easy vocabulary words for young children and English language learners. Yellow cells at the bottom of each list indicate the new items. Final stimulus lists were 84 items long.*

|  | Mean RT | SD RT | Mean % correct | SD % correct |
| --- | --- | --- | --- | --- |
| Pseudo Word | 0.934 | 0.266 | 74.3 | 16.4 |
| Real Word | 0.831 | 0.220 | 79.8 | 12.8 |

***Supplementary Table 2. Percent correct and response times for real and pseudo words.*** *Mean and standard deviation is shown for response time (RT) and percent correct*
